# Metabolic Dysfunction and Coronary Plaque Vulnerability: The Predictive Role of Insulin Resistance Indices in Cardiovascular Outcomes

**DOI:** 10.1002/mco2.70636

**Published:** 2026-02-05

**Authors:** Yue Yu, Jiasheng Yin, Weifeng Guo, Han Chen, Changyi Zhou, Chenguang Li, Cheng Yan, Yanli Song, Dijia Wu, Mengsu Zeng, Li Shen, Junbo Ge

**Affiliations:** ^1^ Departmentof Cardiology Zhongshan Hospital, Fudan University, Shanghai Institute of Cardiovascular Diseases Shanghai China; ^2^ State Key Laboratory of Cardiovascular Diseases Zhongshan Hospital Fudan University Shanghai China; ^3^ NHC Key Laboratory of Ischemic Heart Diseases Shanghai China; ^4^ National Clinical Research Center for Interventional Medicine Shanghai China; ^5^ Institutes of Biomedical Sciences Fudan University Shanghai China; ^6^ Departmentof Radiology Zhongshan Hospital Fudan University Shanghai China; ^7^ Shanghai United Imaging Intelligence Shanghai China

**Keywords:** coronary artery disease, coronary computed tomography angiography, high‐risk plaque, insulin resistance

## Abstract

Significant residual cardiovascular risk persists in patients diagnosed with coronary artery disease despite intensive lipid‐lowering therapy. Although insulin resistance (IR) is an established epidemiological risk factor, the biological mechanisms by which it promotes plaque destabilization remain poorly understood. This single‐center retrospective study, involving 1271 patients, investigated the relationships between four validated IR indices—triglyceride‐glucose (TyG), TyG–body mass index (TyG–BMI), metabolic score for insulin resistance (METS‐IR), and atherogenic index of plasma (AIP)—and high‐risk coronary plaque characteristics quantified by coronary computed‐tomography angiography. Patients with coronary atherosclerosis demonstrated significantly higher IR indices than plaque‐free controls, with all indices exhibiting strong correlations with a high‐risk plaque burden. During follow‐up, 41 patients experienced major adverse cardiovascular events (MACEs), and higher TyG index, AIP, and METS–IR independently predicted MACE after multivariable adjustment, whereas TyG–BMI exhibited a similar but non‐significant trend. A composite model integrating high‐risk plaque burden, pericoronary fat attenuation index, and the four IR indices achieved superior prognostic accuracy, substantially outperforming individual biomarkers. These findings provide novel mechanistic insights into how metabolic dysfunction promotes coronary plaque vulnerability and identify a promising integrated approach for residual risk stratification in patients with coronary artery disease. In this study, IR indices (TyG, TyG‐BMI, AIP, and METS‐IR) correlated high‐risk coronary plaque features in 1271 patients. During 48‐month follow‐up, all indices independently predicted MACEs. Combined with coronary imaging markers, the composite model achieved AUC 0.82, revealing metabolic dysfunction drives plaque destabilization in coronary disease.

## 1 Introduction

Coronary artery disease (CAD) is the leading cause of mortality worldwide. In 2021, 20.5 million deaths globally were attributed to cardiovascular diseases, representing approximately one‐third of all deaths [1]. Although remarkable therapeutic advances in lipid management, particularly high‐intensity statin therapy, have significantly improved patient outcomes, residual cardiovascular risk persists, even in patients who achieve guideline‐recommended low‐density lipoprotein cholesterol (LDL–C) target levels. Large‐scale clinical trials have consistently demonstrated that this residual risk encompasses a complex interplay of non‐lipid factors, including dysregulated triglyceride metabolism, cholesterol remnant particles, systemic inflammation, and underlying metabolic dysfunction, highlighting the critical need to identify and target alternative pathophysiological pathways [2, 3, 4].

Insulin resistance (IR), characterized by decreased tissue responsiveness to insulin stimulation, has emerged as a pivotal mechanistic link between metabolic disorders and atherosclerotic cardiovascular disease [5, 6, 7]. IR is a core defect in cardiometabolic diseases and promotes atherosclerosis through multiple intertwined pathophysiological pathways. These include endothelial dysfunction, enhanced inflammatory responses, increased oxidative stress, altered lipid metabolism with elevated remnant lipoproteins, and impaired bioavailability of nitric oxide [8, 9]. The pervasive nature of IR underscores its potential role in driving the residual risk observed in patients treated with statins.

The clinical significance of IR has been further validated by the development of readily accessible surrogate indices. For example, the TG‐glucose index (TyG) has demonstrated robust predictive utility, with a meta‐analysis reporting that patients in the highest TyG index category had a 2.09‐fold greater risk for major adverse cardiovascular events (MACEs) after acute coronary syndrome [10]. Other indices, such as the TyG–body mass index (TyG–BMI), metabolic score for insulin resistance (METS–IR), and the atherogenic index of plasma (AIP), have also exhibited independent associations with incident cardiovascular disease [11, 12]. Furthermore, emerging evidence suggests that IR indices are correlated with specific plaque characteristics. An elevated TyG index is associated with increased coronary artery calcification progression and vulnerable plaques in patients with type 2 diabetes mellitus [13, 14]. Moreover, when combined with established risk scores, the TyG index significantly enhanced risk stratification, improving the predictive accuracy of the GRACE score, from 0.735 to 0.744 [15].

However, the comparative utility of various IR indices in reflecting high‐risk coronary plaque (HRP) features and their integrated prognostic utility when combined with coronary computed tomographic angiography (CCTA)–derived markers, such as pericoronary inflammation, remains unexplored. As such, the present study aimed to investigate the associations between four distinct IR indices and coronary plaque characteristics and evaluate their collective prognostic utility for predicting MACE in patients diagnosed with CAD.

## 2 Results

### 2.1 Baseline Characteristics

**FIGURE 1:**
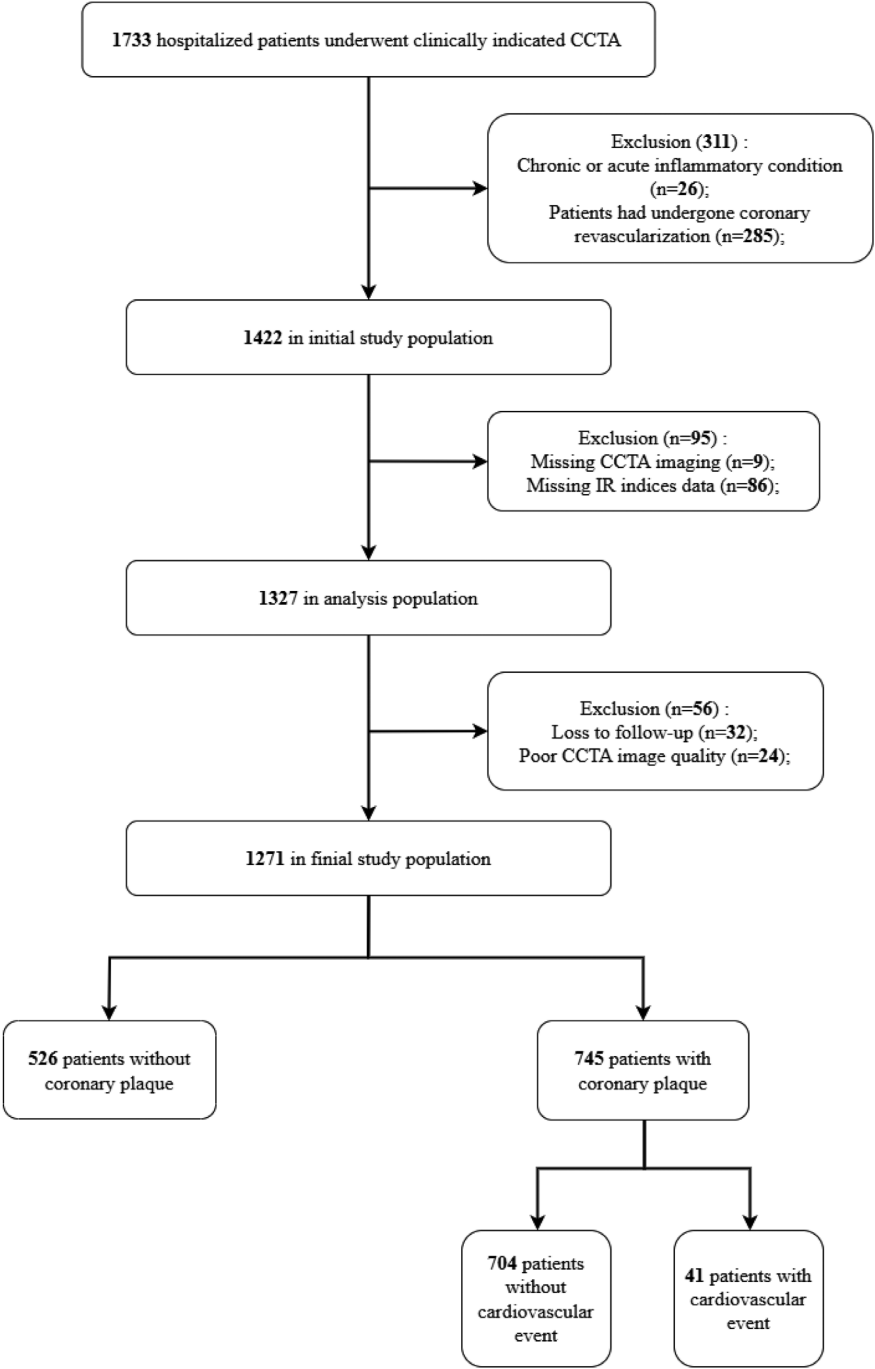
Flowchart of the study design. CCTA, coronary computed tomography angiography; IR, insulin resistance.

After applying these criteria, data from 1271 patients were included in the final analysis (Figure 1). Data from 1271 patients were included according to predefined inclusion and exclusion criteria, 745 (58.62%) of whom were diagnosed with coronary atherosclerosis. The demographic and clinical characteristics of the entire study cohort are summarized in Table S1. The mean (± SD) age of the cohort was 61.24 ± 13.44 years, with a male predominance (*n* = 734 [57.80%]). The mean BMI was 25.71 ± 16.48 kg/m^2^, left ventricular ejection fraction was 61.90 ± 12.57%, and fat attenuation index (FAI) was −79.10 ± 7.79 Hounsfield units (HU). Patients with coronary atherosclerosis exhibited significantly higher prevalence of traditional cardiovascular risk factors, including hypertension, diabetes mellitus, and a history of smoking than those without atherosclerosis. Notably, there were no significant differences in LDL‐C levels or PCSK9 inhibitor use between the two groups.

Further analysis focused on the 745 patients with confirmed coronary atherosclerosis (Table 1). In this subgroup, the mean age was 65.21 ± 12.18 years, with 469 (63.04%) male patients. The mean number of HRPs was 1.35 ± 1.41, and the mean FAI value was −78.30 ± 7.57 HU. When comparing patients who experienced cardiovascular events with those who remained event free, no statistically significant differences were observed in sex distribution, BMI, prevalence of hypertension, smoking status, LDL–C level, or use of antiplatelet agents, statins, or PCSK9 inhibitors.

**TABLE 1:** Baseline characteristics according to MACE.

	Total (*n* = 745)	No cardiovascular events (*n* = 704)	Cardiovascular events (*n* = 41)	*p* value
Age, years	65.21 ± 12.18	64.93 ± 12.17	69.90 ± 11.58	0.0145
Male, *n* (%)	469 (63.04%)	439 (62.45%)	30 (73.17%)	0.167
BMI, kg/m^2^	26.13 ± 18.56	26.20 ± 19.08	25.07 ± 3.54	0.95
Hypertension, *n* (%)	503 (67.52%)	473 (67.19%)	30 (73.17%)	0.4268
Diabetes mellitus, *n* (%)	242 (32.48%)	226 (32.10%)	16 (39.02%)	0.3579
Smoking, *n* (%)	131 (17.58%)	122 (17.33%)	9 (21.95%)	0.1328
Low‐density lipoprotein, mmol/L	2.35 ± 0.84	2.36 ± 0.84	2.14 ± 0.75	0.1428
High‐density lipoprotein cholesterol, mg/dL	47.31 ± 12.30	47.74 ± 12.27	39.84 ± 10.40	0.0001
Triglycerides, mg/dL	146.25 ± 96.65	146.00 ± 97.62	150.63 ± 79.05	0.3484
Fasting plasma glucose, mg/dL	104.66 ± 36.41	103.76 ± 36.11	120.10 ± 38.41	<0.0001
Glycated hemoglobin, %	6.24 ± 1.25	6.22 ± 1.21	6.58 ± 1.73	0.2407
hs‐CRP, mg/L	2.22 ± 11.79	2.03 ± 11.75	5.61 ± 12.16	0.0082
White blood cell count, ×10^9^/L	6.03 ± 1.76	6.01 ± 1.77	6.29 ± 1.56	0.1136
Aspirin, *n* (%)	359 (48.19%)	337 (47.87%)	22 (53.66%)	0.4711
P2Y_12_ inhibitor, *n* (%)	259 (34.77%)	242 (34.38%)	17 (41.46%)	0.3545
Statin therapy, *n* (%)	547 (73.42%)	516 (73.30%)	31 (75.61%)	0.7445
PCSK9i therapy, *n* (%)	4 (0.54%)	4 (0.57%)	0 (0.00%)	0.6286
SGLT2 inhibitor therapy, *n* (%)	65 (8.72%)	62(8.81%)	3 (7.32%)	0.7426
LVEF, %	61.00 ± 11.49	61.42 ± 11.07	53.75 ± 15.67	0.0001
Left ventricular diastolic dysfunction, *n* (%)	461 (72.03%)	433 (71.57%)	28 (80.00%)	0.2804
IR indices				
TyG index	8.74 ± 0.66	8.73 ± 0.66	8.95 ± 0.59	0.0280
TyG‐BMI	229.12 ± 164.83	229.35 ± 169.31	225.15 ± 39.33	0.3836
AIP	0.43 ± 0.31	0.43 ± 0.31	0.54 ± 0.29	0.0153
METS‐IR	40.20 ± 29.39	40.14 ± 30.18	41.11 ± 7.84	0.0428
Computed tomography parameters				
High‐risk plaque numbers, *n*	1.35 ± 1.41	1.29 ± 1.35	2.39 ± 1.91	<0.0001
HRP ratio, %	0.48 ± 0.39	0.47 ± 0.39	0.55 ± 0.34	0.1495
FAI, HU	−78.30 ± 7.57	−78.54 ± 7.54	−74.11 ± 6.88	0.0003
Calcified plaque volume, mm^3^	67.96 ± 136.07	63.27 ± 129.90	148.62 ± 201.96	0.0014
Fibrotic plaque volume, mm^3^	105.57 ± 155.77	99.07 ± 147.88	217.17 ± 231.13	<0.0001
Fibrous fatty plaque volume, mm^3^	93.99 ± 118.94	89.60 ± 115.96	169.30 ± 143.74	<0.0001
Low attenuation plaque volume, mm^3^	22.15 ± 33.46	21.62 ± 33.42	31.39 ± 33.20	0.0080
Noncalcified plaque volume, mm^3^	221.71 ± 278.38	210.28 ± 268.50	417.85 ± 364.13	<0.0001

*Note*: Data are presented as mean ± standard deviation (SD) or number (percentage), as appropriate. The bold figures showed significant difference in statistics.

Abbreviations: AIP, atherogenic index of plasma; BMI, body mass index; FAI, fat attenuation index; HDL‐C, high‐density lipoprotein cholesterol; HRP, high‐risk plaque; hsCRP, high‐sensitivity C‐reactive protein; HU, Hounsfield unit; IR, insulin resistance; LDL‐C, low‐density lipoprotein cholesterol; LVEF, left ventricular ejection fraction; MACE, major adverse cardiovascular event; METS‐IR, metabolic score for insulin resistance; PCSK9i, proprotein convertase subtilisin/kexin type 9 inhibitor; SGLT2, sodium–glucose cotransporter 2; TyG, triglyceride–glucose; WBC, white blood cell.

### 2.2 Association Between IR Indices, HRP Features, and Coronary Atherosclerosis

Comparative analysis of IR indices between patients stratified according to the presence of coronary plaques revealed significant differences (Table S1). Patients without coronary atherosclerotic plaques exhibited significantly lower IR indices compared to those with coronary atherosclerotic plaques: TyG index (8.62 ± 0.61 versus [vs.] 8.74 ± 0.66; *p* = 0.0006), TyG–BMI (217.05 ± 114.07 vs. 229.12 ± 164.83; *p* < 0.0001), AIP (0.38 ± 0.32 vs. 0.43 ± 0.31, *p* = 0.0005), and METS–IR (38.13 ± 18.65 vs. 40.20 ± 29.39; *p* = 0.0001). These findings substantiate the role of IR in the progression of coronary atherosclerosis.

**FIGURE 2:**
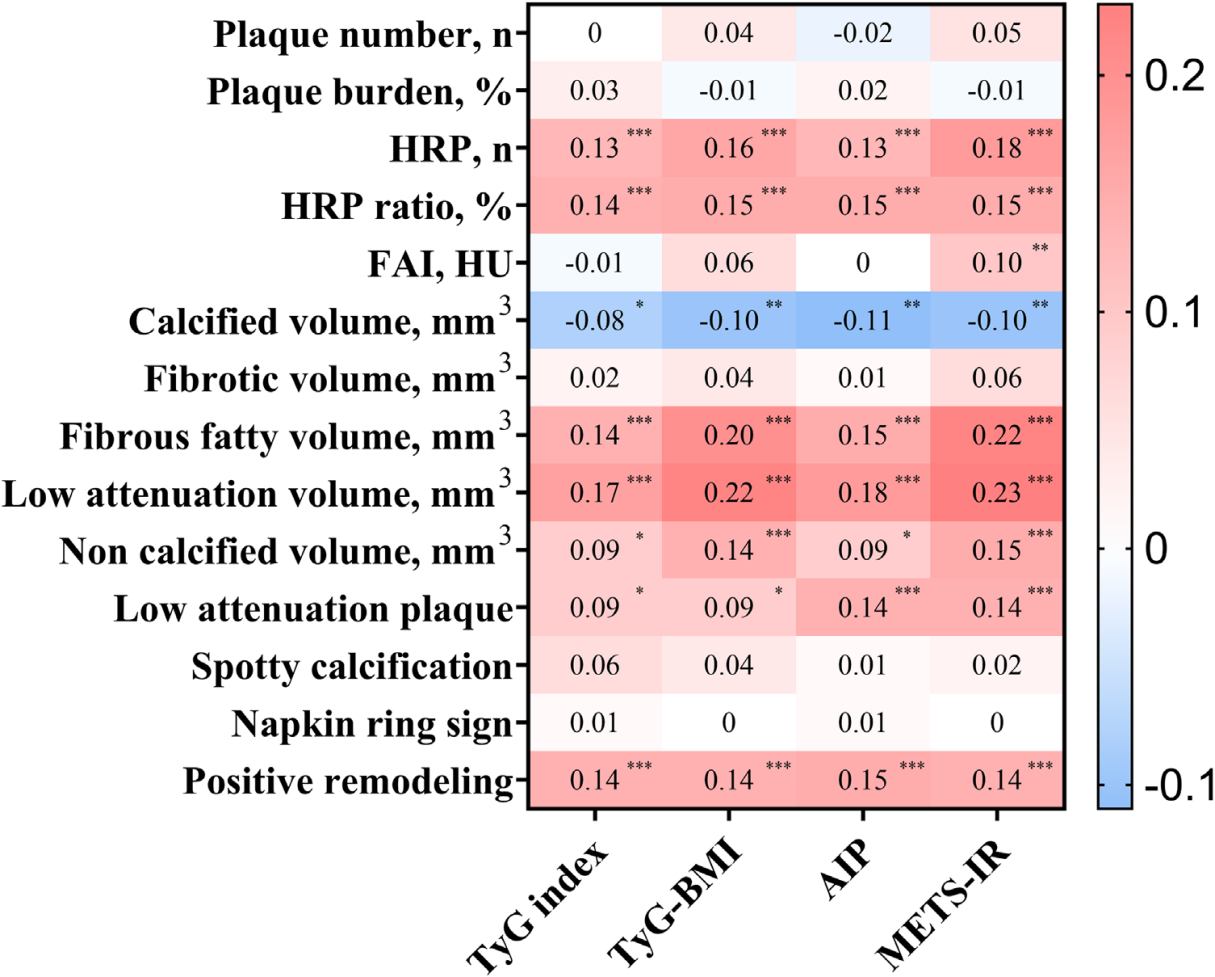
Correlation of IR indices and plaque characteristics. FAI, fat attenuation index; HRP, high‐risk plaque; TyG index, triglyceride‐glucose index; TyG‐BMI, TyG‐body mass index; AIP, atherogenic index of plasma; METS‐IR, metabolic score for insulin resistance. *p < 0.05; **p < 0.001, ***p < 0.0001.

Correlations between the IR indices and coronary plaque characteristics are reported in Figure 2. All IR metrics exhibited significant positive associations with the number of HRPs, including the TyG index (*r* = 0.13, *p* < 0.0001), TyG–BMI (*r* = 0.16, *p* < 0.0001), AIP (*r* = 0.13, *p* < 0.0001), and METS–IR (*r* = 0.18, *p* < 0.0001). Similarly, positive correlations were observed between IR indices and HRP ratio: TyG (*r* = 0.14, *p* < 0.0001), TyG–BMI (*r* = 0.15, *p* < 0.0001), AIP (*r* = 0.15, *p* < 0.0001), and METS–IR (*r* = 0.15, *p* < 0.0001). Regarding the compositional characteristics of plaques, all IR indices (i.e., TyG index, TyG–BMI, AIP, and METS–IR) were positively associated with fibrous fatty plaque volume, low‐attenuation plaque (LAP) volume, and non‐calcified plaque volume, suggesting that IR may be closely associated with the early stage of plaque formation. However, among the IR indices evaluated, only METS–IR exhibited a significant positive correlation with FAI (*r* = 0.10, *p* = 0.0057).

At baseline, patients who subsequently developed MACE exhibited a more adverse cardiometabolic profile and a greater burden of HRPs (Table 1). Patients who experienced cardiovascular events during follow‐up exhibited significantly elevated baseline TyG index (8.73 ± 0.66 vs. 8.95 ± 0.59; *p* = 0.0280), AIP (0.43 ± 0.31 vs. 0.54 ± 0.29; *p* = 0.0153), METS–IR (40.14 ± 30.18 vs. 41.11± 7.84; *p* = 0.0428), number of HRPs (1.29 ± 1.35 vs. 2.39 ± 1.91; *p* < 0.001), and FAI (−78.54 ± 7.54 vs. −74.11 ± 6.88; *p* = 0.0003) compared with those who were event free. Notably, TyG–BMI demonstrated no significant intergroup difference (229.35 ± 169.31 vs. 225.15 ± 39.33; *p* = 0.3836).

**FIGURE 3:**
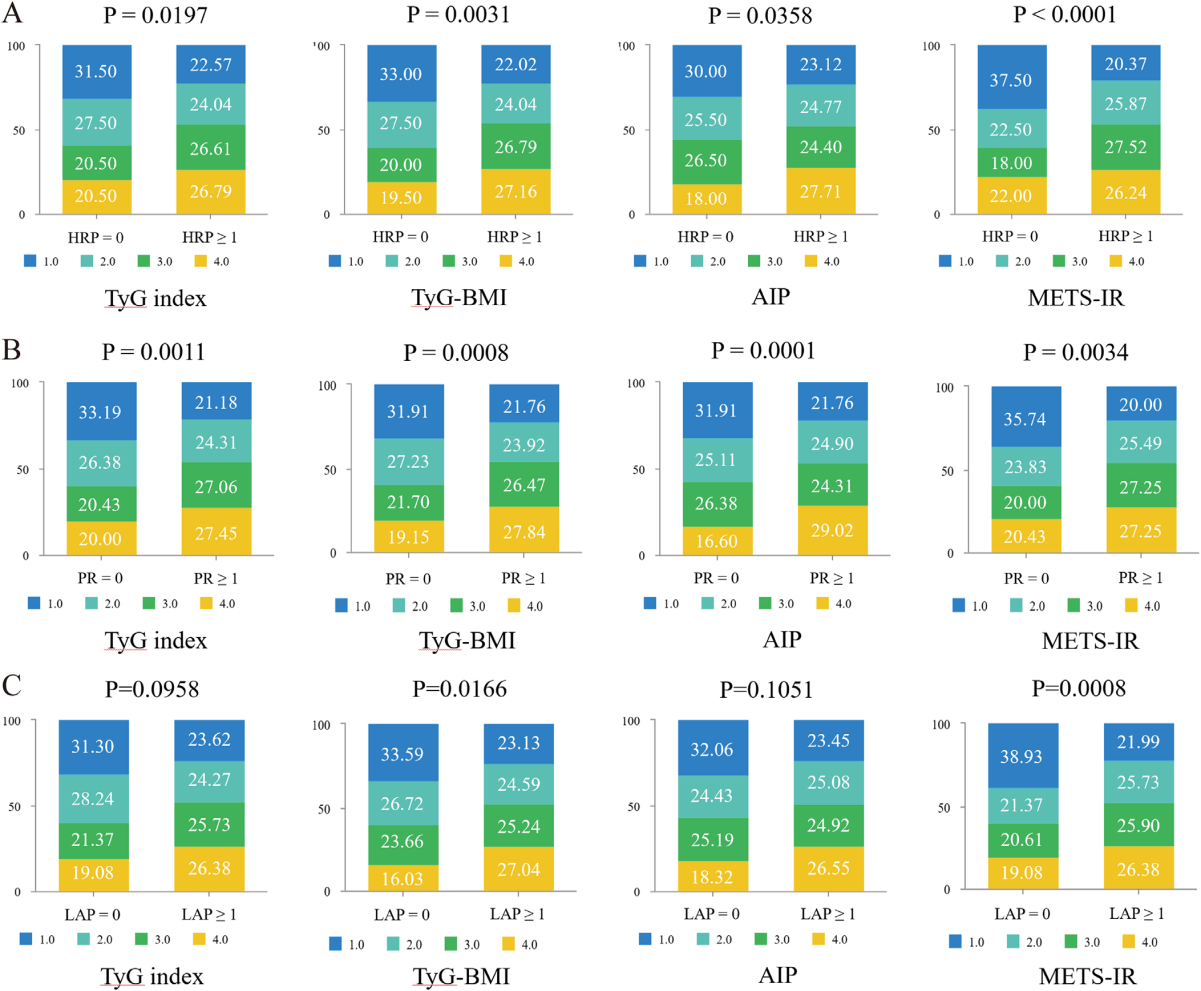
Distribution of HRP and HRP features according to IR indices. (A) The number of HRP was significantly different according to IR indices. (B) PR features were significantly different according to IR indices. (C) LAP features showed significant associations with TyG‐BMI and METS‐IR indices, whereas no significant associations were observed with TyG index and AIP. AIP, atherogenic index of plasma; LAP, low‐attenuation plaque; METS‐IR, metabolic score for insulin resistance; PR, positive remodeling; TyG index, triglyceride‐glucose index; TyG‐BMI, TyG‐body mass index.

Analysis of the morphological features of the HRPs revealed that all IR indices were positively correlated with LAP and positive remodeling (PR). However, no significant associations were observed between IR indices and spotty calcification (SC) or napkin‐ring sign (NRS). Patients with elevated TyG, TyG–BMI, AIP, and METS–IR indices demonstrated increased proportions of HRPs (Figure 3A) and PR (Figure 3B). Notably, only elevated TyG–BMI and METS–IR were associated with a higher proportion of patients with LAP (Figure 3C). None of the IR indices exhibited significant associations with SC or NRS (Figure S1).

Collectively, these findings demonstrated a significant positive association between IR indices and HRP characteristics, suggesting a potential mechanistic link between metabolic dysfunction and coronary plaque vulnerability, which may contribute to adverse cardiovascular outcomes.

### 2.3 Association Between IR Indices and MACEs in CAD Patients

**FIGURE 4:**
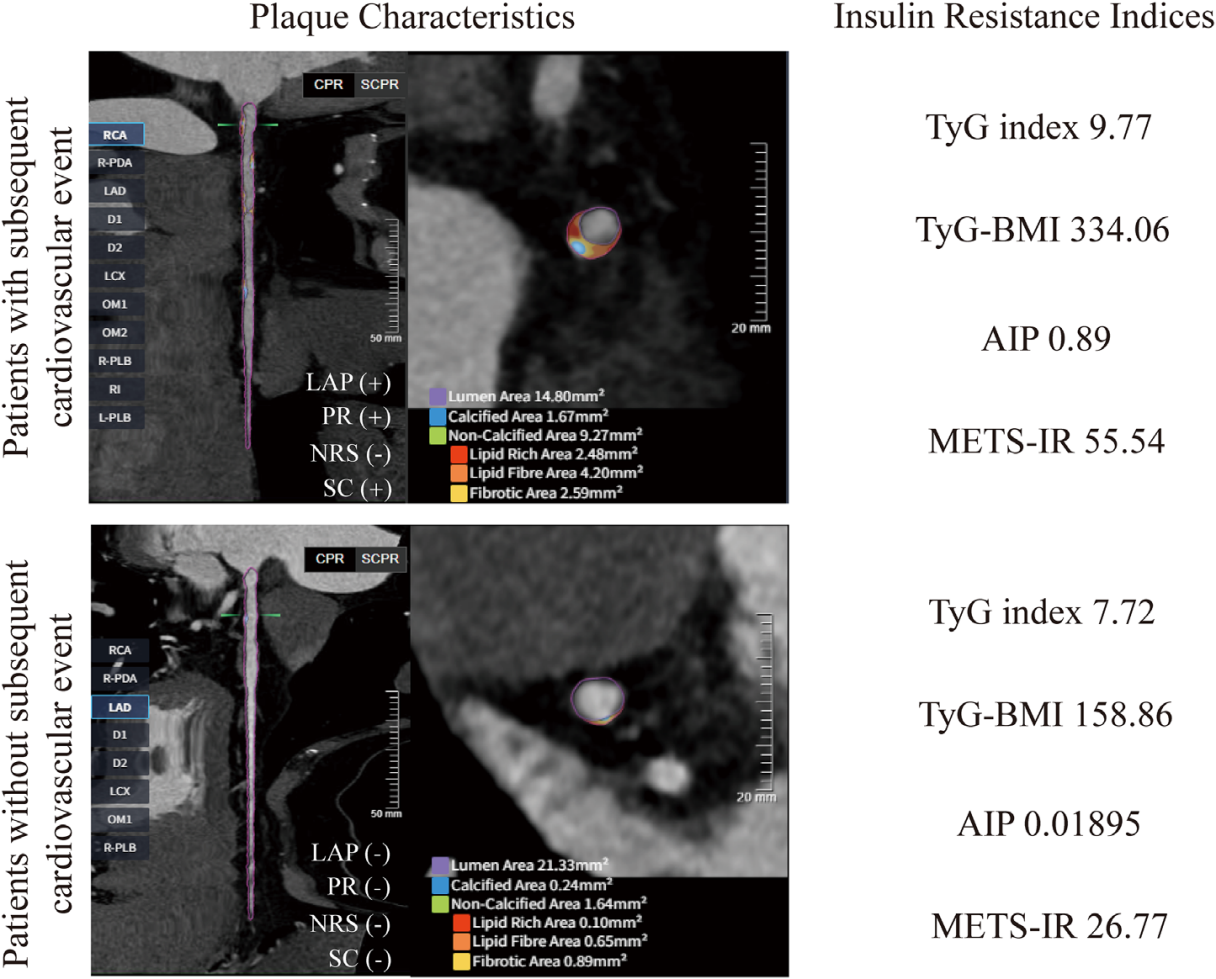
Representative case with and without subsequent cardiovascular disease events. Upper panel: patient who experienced MACE during follow‐up, with elevated IR indices and multiple HRP features on CCTA, including PR, LAP, SC, and NRS. Lower panel: Patient who remained free of MACE, with lower IR indices and absence of HRP features. AIP, atherogenic index of plasma; LAP, low‐attenuation plaque; METS‐IR, metabolic score for insulin resistance; NRS, napkin‐ring sign; PR, positive remodeling; SC, spotty calcification; TyG‐BMI, TyG‐body mass index; TyG index, triglyceride‐glucose index.

During follow‐up, 41 (5.50%) individuals experienced MACE, including eight cardiac deaths, six nonfatal myocardial infarctions, five nonfatal strokes, 18 hospitalizations for unstable angina, and four cases of acute heart failure. Representative images illustrating the IR indices, FAI, and HRP assessed using CCTA in individuals with and without subsequent cardiovascular events are presented in Figure 4.

Cox proportional hazard modeling revealed that higher levels of several IR indices were associated with an increased risk for MACE. In fully adjusted analyses (Model 3), the hazard ratios (HRs) for the highest versus lowest quartile were 3.95 (95% confidence interval [CI] 1.14–13.70; *p* = 0.0307) for the TyG index, 7.40 (95% CI 2.17–25.23; *p* = 0.0014) for AIP, and 4.46 (95% CI 1.26–15.82; *p* = 0.0205) for METS–IR, whereas the corresponding HR for TyG–BMI was 3.25 (95% CI 0.89–11.90; *p* = 0.0756) (Table 2). Restricted cubic spline analyses further characterized these relationships, revealing approximately linear dose–response associations between both the TyG index and AIP and the risk for MACE (*p* for overall = 0.017 and 0.009; *p* for nonlinear = 0.502 and 0.341, respectively). In contrast, no statistically significant overall trends were observed for TyG–BMI (*p* for overall = 0.529) or METS–IR (*p* for overall = 0.196), suggesting that their prognostic impact may be limited to extreme values within the cohort (Figure S2).

**TABLE 2:** Association between IR index and MACE.

	Model 1		Model 2		Model 3	
	HR [95%CI]	*p*	HR [95%CI]	*p*	HR [95%CI]	*p*
TyG						
Q1	Ref.		Ref.		Ref.	
Q2	1.41 (0.50–3.97)	0.5134	1.69 (0.60–4.79)	0.3205	0.88 (0.21–3.67)	0.8584
Q3	1.51 (0.54–4.24)	0.4361	2.00 (0.70–5.71)	0.1937	3.05 (0.87–10.69)	0.0809
Q4	2.79 (1.10–7.08)	0.0306	4.11 (1.58–10.70)	0.0037	3.95 (1.14–13.70)	0.0307
TyG‐BMI						
Q1	Ref.		Ref.		Ref.	
Q2	1.28 (0.52–3.19)	0.6064	1.41 (0.56–3.51)	0.4631	2.11 (0.61–7.31)	0.2394
Q3	0.85 (0.31–2.34)	0.7364	0.98 (0.35–2.72)	0.9701	1.78 (0.49–6.54)	0.3819
Q4	1.85 (0.79–4.37)	0.1646	2.83 (1.16–6.90)	0.0219	3.25 (0.89–11.90)	0.0756
AIP						
Q1	Ref.		Ref.		Ref.	
Q2	2.63 (0.84–8.25)	0.0984	3.43 (1.08–10.87)	0.0361	2.66 (0.74–9.56)	0.1334
Q3	2.49 (0.78–7.95)	0.1223	3.49 (1.08–11.28)	0.0370	3.76 (1.05–13.47)	0.0418
Q4	3.82 (1.28–11.44)	0.0164	6.54 (2.11–20.26)	0.0011	7.40 (2.17–25.23)	0.0014
METS‐IR						
Q1	Ref.		Ref.		Ref.	
Q2	0.97 (0.36–2.58)	0.9502	1.05 (0.39–2.82)	0.9186	1.46 (0.39–5.44)	0.5756
Q3	1.07 (0.41–2.78)	0.8883	1.30 (0.50–3.40)	0.5963	1.56 (0.41–5.98)	0.4931
Q4	2.01 (0.86–4.70)	0.1061	2.98 (1.23–7.25)	0.0158	4.46 (1.26–15.82)	0.0205

*Note*: Data are presented as HR with 95% CI. Q1∼Q4 indicate increasing quartiles of each IR index, with Q1 used as the reference category. Model 1: unadjusted. Model 2: adjusted for age and sex. Model 3: further adjusted for hypertension, diabetes mellitus, current smoking, left ventricular ejection fraction, left ventricular diastolic dysfunction, statin therapy, antiplatelet therapy, and LDL‐C. The bold figures showed significant difference in statistics.

Abbreviations: AIP, atherogenic index of plasma; CI, confidence interval; HR, hazard ratio; IR, insulin resistance; LDL‐C, low‐density lipoprotein cholesterol; MACE, major adverse cardiovascular event; METS‐IR, metabolic score for insulin resistance; TyG, triglyceride–glucose index; TyG‐BMI, triglyceride–glucose–body mass index.

**FIGURE 5:**
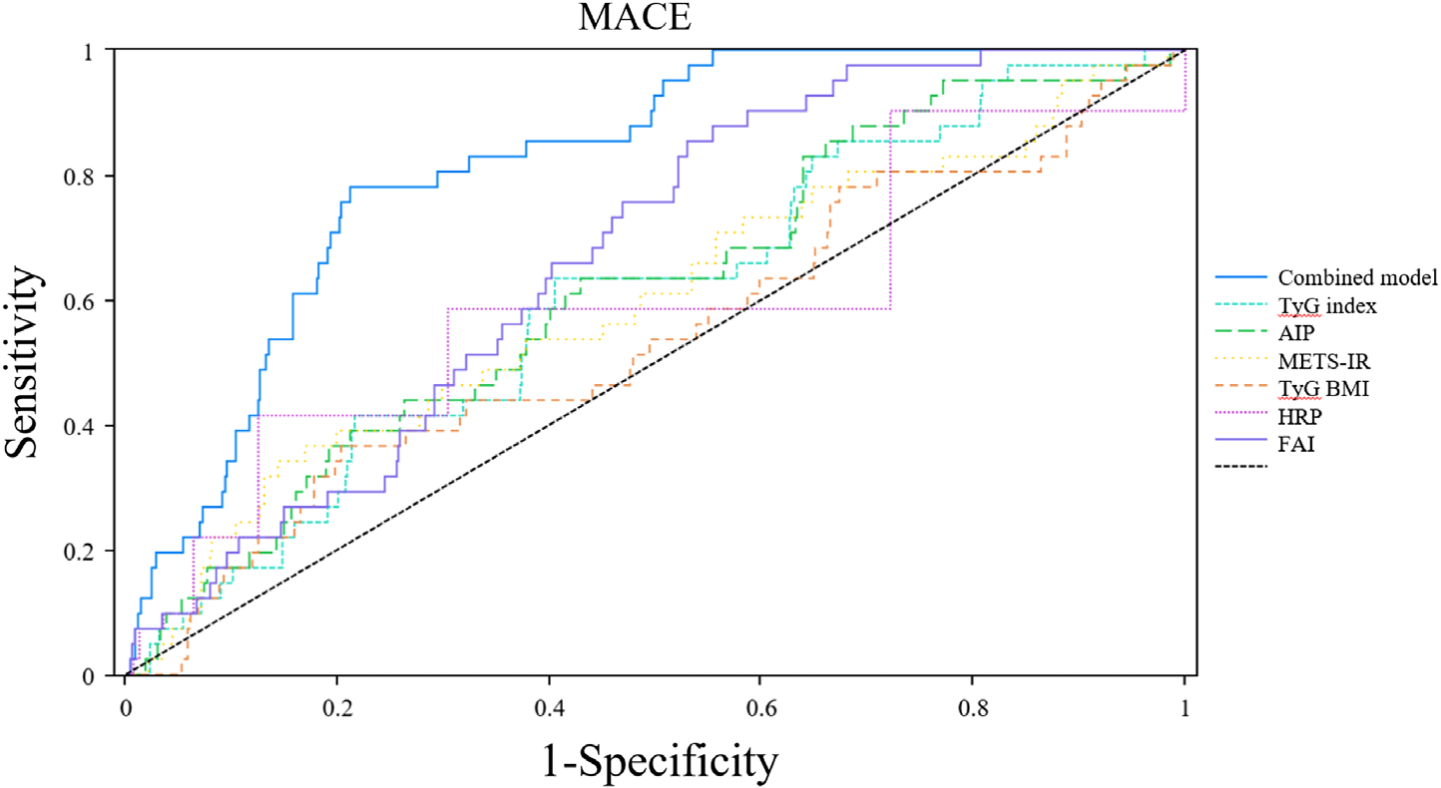
ROC curves for predictive MACE. MACE prediction performance. AIP, atherogenic index of plasma; FAI, fat attenuation index; HRP, high‐risk plaque; MACE, major adverse cardiac events; METS‐IR, metabolic score for insulin resistance; TyG‐BMI, TyG‐body mass index; TyG index, triglyceride‐glucose index.

Table 3 and Figure 5 report the comparative predictive performances of different metabolic indices for MACE, in which individual biomarkers exhibited varying discriminative abilities (TyG–BMI: area under the receiver operating characteristic curve [AUC] 0.54, *p* = 0.3836; HRP: AUC 0.69, *p* < 0.0001; FAI: AUC 0.67, *p* = 0.0003; AIP: AUC 0.61, *p* = 0.0153; TyG index: AUC 0.60, *p* = 0.0280; METS–IR: AUC 0.59, *p* = 0.0428). Notably, the combined model integrating HRP, FAI, and IR indices achieved superior predictive accuracy, with an AUC of 0.82 (95% CI 0.77–0.87; *p* < 0.0001), substantially outperforming all individual biomarkers and highlighting the clinical utility of multiparameter approaches for cardiovascular risk stratification.

**TABLE 3:** Predictive performance of insulin resistance indices and CT‐derived parameters for MACE.

	AUC	95% CI	*p*
TyG index	0.60	0.52–0.69	0.0280
TyG‐BMI	0.54	0.44–0.64	0.3836
AIP	0.61	0.53–0.70	0.0153
METS‐IR	0.59	0.50–0.69	0.0428
HRP	0.69	0.60–0.78	<0.0001
FAI	0.67	0.60–0.74	0.0003
Combined model (HRP+FAI+ IR index)	0.82	0.77–0.87	<0.0001

## 3 Discussion

Leveraging CTTA to characterize HRP morphology and pericoronary adipose tissue inflammation, results of our study demonstrate that routinely available IR indices are strongly associated with coronary plaque vulnerability and, for several indices, with subsequent clinical events. All four IR indices were higher in patients with coronary atherosclerosis and correlated with HRP burden, whereas a higher TyG index, AIP, and METS–IR independently predicted MACE, with TyG–BMI exhibiting a similar but non‐significant trend. Collectively, these findings delineate a mechanistic pathway linking systemic metabolic dysfunction, vulnerable plaque phenotypes, and residual cardiovascular risk in established CAD.

Previous studies have consistently linked IR to adverse coronary outcomes [16, 17, 18], with the TyG index emerging as a particularly robust surrogate in acute coronary syndrome cohorts [19, 20]. Similarly, TyG–BMI, AIP, and METS–IR have exhibited independent prognostic utility for cardiovascular events [11, 21, 22]. However, the precise biological mechanisms connecting systemic metabolic dysfunction to plaque destabilization remain elusive. By leveraging CCTA for comprehensive plaque phenotyping encompassing HRP burden, compositional traits, and pericoronary inflammation, we demonstrated that each IR index correlated strongly with HRP count and proportion, and detailed plaque metrics. Together with previous reports addressing IR–plaque associations, our findings underscore a more nuanced, multifactorial interplay between IR and plaque vulnerability than has been understood to date.

The divergent predictive performances of the four IR indices reflect their distinct pathobiological underpinnings. Among them, AIP exhibited the strongest association with MACE (HR 7.40 for the highest vs. lowest quartile in the fully adjusted model), plausibly because its TG‐to‐high‐density lipoprotein cholesterol (HDL‐C) formulation directly reflects the balance between proatherogenic and cardioprotective lipoproteins. Similarly, the TyG index exhibited a robust association with events, and our dose–response analyses indicated that higher TyG and AIP values were consistently related to greater MACE risk across the clinically observed range. METS–IR, which integrates glucose, triglyceride, BMI, and HDL‐C levels, was also correlated with HRP burden and was independently associated with MACE among patients with the highest METS–IR levels; however, its association with events appeared less progressive across intermediate ranges. Clinically, this pattern suggests that METS–IR may primarily capture excess risk in patients with more advanced metabolic disturbances and that larger cohorts will be required to determine whether more modest elevations carry incremental prognostic utility. Notably, BMI‐containing indices (i.e., TyG–BMI and METS–IR) were the only indices associated with pericoronary FAI, supporting the concept that obesity‐related inflammation enhances the detection of lipid‐rich vulnerable plaque components. Collectively, these observations reveal that different composite IR metrics capture partially overlapping—but non‐identical—aspects of the metabolic–inflammatory axis driving coronary plaque vulnerability.

IR accelerates the accumulation of lipid‑rich, noncalcified plaques, thereby destabilizing atherosclerotic lesions and increasing the risk for acute coronary events. Mechanistically, IR impairs vascular smooth‑muscle‑cell survival and proliferation by attenuating anti‑apoptotic/pro‑proliferative insulin signaling while amplifying pro‑inflammatory pathways; this imbalance alters collagen metabolism and weakens the fibrous cap, compromising plaque stability [23, 24]. The cellular dysfunction unfolds within a systemic pro‑inflammatory milieu—characterized by elevated cytokines and endothelial adhesion molecules—that promotes monocyte recruitment, macrophage infiltration, and foam‑cell formation, key steps in plaque growth and necrotic‑core expansion [25, 26]. Consistent with these mechanisms, our study revealed that higher IR indices were positively correlated with noncalcified plaque volume, highlighting a preferential link between metabolic dysfunction and early vulnerable plaque phenotypes. These results align with a study by Rokicka et al. [27], who reported similar associations between IR markers and acute myocardial infarction.

The consistent association between the IR indices and HRP features supports their potential role in clinical risk stratification. Because these indices are readily available and inexpensive to calculate, they can be integrated into existing risk assessment protocols to help identify patients who may benefit from more aggressive risk factor modifications, even among those who achieve conventional lipid target levels. Future studies should examine whether tracking these indices over time adds prognostic value.

Our study had several limitations, the first of which was its single‐center design, which may limit the generalizability of our findings to broader populations with different ethnic backgrounds, high‐risk populations, and clinical characteristics. Second, while we used comprehensive non‐insulin‐based indices to assess IR, we did not use direct measures of insulin sensitivity, such as the hyperinsulinemic‐euglycemic clamp technique, which remains the gold standard but is impractical for large‐scale studies. Finally, our findings require external validation in diverse populations to confirm reproducibility and clinical applicability. Future prospective studies with larger and more heterogeneous cohorts and longer follow‐up periods are needed to validate these findings and explore the potential therapeutic implications of targeting IR for cardiovascular risk reduction.

## 4 Conclusion

In conclusion, IR indices were significantly associated with HRP characteristics and functioned as independent predictors of MACE in patients with CAD. Integrating these metabolic markers with CCTA‐derived plaque metrics improves prognostic discrimination beyond conventional risk factors and deepens the current mechanistic understanding of how metabolic dysfunction drives coronary plaque instability.

## 5 Methods

### 5.1 Study Population

This single‐center retrospective study screened patients from January 1, 2018, to December 31, 2023. The inclusion criteria were as follows: hospitalization with CCTA; absence of cancer, acute or chronic inflammation, and autoimmune diseases; no prior coronary revascularization; and availability of analyzable plaque features and IR indices.

### 5.2 Calculation of IR Indices

Four validated IR indices were calculated using standardized equations based on fasting laboratory parameters.

### 5.3 CCTA Image Acquisition and Analysis

CCTA examinations were performed using a 256‐slice CT scanner in accordance with standardized institutional protocols, with all patients receiving appropriate premedication with beta‐blockers to optimize image quality. Plaque quantification and FAI extraction were performed using a locked deep‑learning pipeline (uAI‑CoronaryCTA, version 3.0; United Imaging, Shanghai, China), in which image quality assessment was performed systematically and examinations with non‐diagnostic quality due to motion artifacts, severe calcification, or inadequate contrast enhancement were excluded from analysis [28, 29].

Coronary plaque burden was quantified as the percentage ratio of cross‐sectional plaque area to total vessel area at the site of maximum stenosis, and four established adverse plaque characteristics were systematically evaluated including PR defined as a remodeling index (lesion diameter/reference diameter) ≥ 1.1, LAP characterized by focal plaque components with CT attenuation < 30 HU, SC identified as discrete calcified deposits < 3 mm in any dimension within the plaque, and NRS defined as a ring‐like pattern of high‐attenuation plaque surrounding a central core of low‐attenuation material. HRP was defined by the presence of ≥ 2 adverse plaque characteristics, and patients were categorized as having adverse plaque morphology if ≥ 1 adverse plaque feature(s) were identified in any coronary vessel [30, 31].

Pericoronary adipose tissue inflammation was quantified using the FAI, with analysis performed on a standardized 40 mm proximal segment of each major coronary artery. Pericoronary adipose tissue was defined by voxel attenuation values ranging from −190 to −30 HU [32, 33].

### 5.4 Patient Follow‐Up and Adjudication of Clinical Events

All patients enrolled completed the study protocol with a comprehensive clinical follow‐up, with a median follow‐up of 48 months (interquartile range, 36–60 months). Follow‐up assessments were performed through scheduled outpatient clinic visits, structured telephone interviews, and electronic health record reviews. The primary composite endpoint was MACE, defined as the occurrence of cardiac death, non‐fatal myocardial infarction, or hospitalization for unstable angina or acute heart failure.

### 5.5 Statistical Analysis

Continuous variables are expressed as mean ± standard deviation (SD) for normally distributed data, or median and interquartile range (IQR) for non‐normally distributed data. Group comparisons were performed using an unpaired Student's *t*‐test or the Mann–Whitney *U* test, as appropriate. Categorical variables were compared using the chi‐squared test.

Univariate associations were assessed using Pearson's correlation coefficients for normally distributed variables and Spearman's correlation coefficients, with Bonferroni correction, for non‐normally distributed variables.

Cox proportional hazards regression was performed to estimate HRs and corresponding 95% CIs for group comparisons. Multivariate Cox regression analysis was used to derive the adjusted HRs and 95% CIs. The AUC was calculated to assess the predictive abilities of the four IR indices in combination with HRP and FAI for MACE.

All statistical tests were two‐tailed and differences with *p* < 0.05 were considered to be significant. Analyses were performed using SPSS version 25.0 (IBM Corporation, Armonk, NY, USA) and R version 3.6.2 (R Core Team; R Foundation for Statistical Computing, Vienna, Austria).

## Author Contributions

Junbo Ge, Li Shen, and Mengsu Zeng designed and supervised the experiments. Han Chen, Changyi Zhou, Chenguang Li, Cheng Yan, Yanli Song, and Dijia Wu collected the clinical data. Yue Yu, Jiasheng Yin, and Weifeng Guo analyzed the data and wrote the paper. All authors approved the final manuscript.

## Funding

This work was supported by grants from the National Natural Science Foundation of China (82200377, 82170342, T2288101).

## Ethics Statement

The study was approved by the ethics committee of Zhongshan Hospital, Fudan University and performed in accordance with institutional guidelines (Clinical Trial Registration: ChiCTR2300072219). Written informed consent was obtained from all participants in accordance with the institutional guidelines and the Declaration of Helsinki.

## Conflicts of Interest

The authors declare no conflicts of interest.

## Supporting information




**Supplemental Table 1**: Baseline characteristics according to coronary atherosclerosis
**Supplemental Figure 1**: Distribution of NRS and SC according to IR indices A. NRS features were not significantly different according to IR indices. B. SC features were not significantly different according to IR indices. SC, Spotty calcification; NRS, napkin‐ring sign; TyG index, triglyceride‐glucose index; TyG‐BMI, TyG‐body mass index; AIP, atherogenic index of plasma; METS‐IR, metabolic score for insulin resistance.
**Supplemental Figure 2**: Restricted Cubic Spline Analyses of the Association Between IR Indices and MACE. A. RCS analysis of TyG and MACE; B. RCS analysis of TyG‐BMI and MACE; C. RCS analysis of AIP and MACE; D. RCS analysis of METS‐IR and MACE. TyG index, triglyceride‐glucose index; TyG‐BMI, TyG‐body mass index; AIP, atherogenic index of plasma; METS‐IR, metabolic score for insulin resistance; LVEF, left ventricular ejection fraction; hs‐CRP, hypersensitive C reactive protein; LDL, low‐density lipoprotein.

## Data Availability

The datasets supporting this article are available from the corresponding author on reasonable request.
