# Supplementary material for: Metabolic Dysfunction and Coronary Plaque Vulnerability: The Predictive Role of Insulin Resistance Indices in Cardiovascular Outcomes
Source: MedComm (2020). 2026 Feb 5;7(2):e70636. doi: 10.1002/mco2.70636 (PMC12877314; doi:10.1002/mco2.70636)
Supplement: Supplementary file 1 — Supplemental Table 1: Baseline characteristics according to coronary atherosclerosis Supplemental Figure 1: Distribution of NRS and SC according to IR indices A. NRS features were not significantly different according to IR indices. B. SC features were not significantly different according to IR indices. SC, Spotty calcification; NRS, napkin‐ring sign; TyG index, triglyceride‐glucose index; TyG‐BMI, TyG‐body mass index; AIP, atherogenic index of plasma; METS‐IR, metabolic score for insulin resistance. Supplemental Figure 2: Restricted Cubic Spline Analyses of the Association Between IR Indices and MACE. A. RCS analysis of TyG and MACE; B. RCS analysis of TyG‐BMI and MACE; C. RCS analysis of AIP and MACE; D. RCS analysis of METS‐IR and MACE. TyG index, triglyceride‐glucose index; TyG‐BMI, TyG‐body mass index; AIP, atherogenic index of plasma; METS‐IR, metabolic score for insulin resistance; LVEF, left ventricular ejection fraction; hs‐CRP, hypersensitive C reactive protein; LDL, low‐density lipoprotein. [file MCO2-7-e70636-s001.docx]

**Metabolic Dysfunction and Coronary Plaque Vulnerability: The Predictive Role of Insulin Resistance Indices in Cardiovascular Outcomes**

Yue Yu, MD^1,2,3,4,5#^; Jiasheng Yin, MD ^1,2,3,4,5#^; Weifeng Guo, MS^6#^; Han Chen, PhD^1,2,3,4,5^; Changyi Zhou, PhD^1,2,3,4,5^; Chenguang Li, PhD^1,2,3,4,5^; Cheng Yan^6^; Yanli Song, PhD^7^; Dijia Wu, PhD^7^; Mengsu Zeng, PhD^6*^; Li Shen, PhD ^1,2,3,4,5*^; Junbo Ge, MD^1,2,3,4,5*^

1.Departmentof Cardiology, Zhongshan Hospital, Fudan University, Shanghai Institute of Cardiovascular Diseases, China.

2.State Key Laboratory of Cardiovascular Diseases, Zhongshan Hospital, Fudan University.

3.NHC Key Laboratory of Ischemic Heart Diseases.

4.National Clinical Research Center for Interventional Medicine, Shanghai, China.

5.Institutes of Biomedical Sciences, Fudan University, Shanghai, China.

6.Departmentof Radiology, Zhongshan Hospital, Fudan University, China.

7.Shanghai United Imaging Intelligence, Shanghai, China.

#Yue Yu, Jiasheng Yin, and Weifeng Guo have contributed equally to this work.

* Corresponding authors:

Prof. Junbo Ge: jbge@zs-hospital.sh.cn

Li Shen, PhD: shen.li1@zs-hospital.sh.cn

Mengsu Zeng, PhD: zeng.mengsu@zs-hospital.sh.cn

Supplemental table 1 Baseline characteristics according to coronary atherosclerosis

|  | Total  (n=1271) | No coronary atherosclerosis patients (n=526) | Coronary atherosclerosis patients (n=745) | P Value |
| --- | --- | --- | --- | --- |
| Age, y | 61.24±13.44 | 55.63±13.14 | 65.21±12.18 | <0.0001 |
| Male, n (%) | 734(57.80%) | 265(50.38%) | 469(63.04%) | <0.0001 |
| BMI, kg/m^2^ | 25.71±16.48 | 25.11±12.97 | 26.13±18.56 | 0.0002 |
| Hypertension, n (%) | 728(57.28%) | 225(42.78%) | 503(67.52%) | <0.0001 |
| Diabetes mellitus, n (%) | 325(25.57%) | 83(15.78%) | 242(32.48%) | <0.0001 |
| Smoking, n (%) | 193(15.18%) | 62(11.79%) | 131(17.58%) | 0.0020 |
| Low-density lipoprotein cholesterol, mmol/L | 2.41±0.83 | 2.45±0.80 | 2.38±0.84 | 0.1205 |
| High-density lipoprotein cholesterol, mg/dL | 48.45±12.61 | 50.06±12.87 | 47.31±12.30 | <0.0001 |
| Triglycerides, mg/dL | 142.60±96.93 | 137.43±97.19 | 146.25±96.65 | 0.0144 |
| Fasting plasma glucose, mg/dL | 99.85±32.50 | 93.05±24.44 | 104.66±36.41 | <0.0001 |
| Glycated haemoglobin, % | 6.03±1.08 | 5.73±0.70 | 6.24±1.25 | <0.0001 |
| Aspirin, n (%) | 514(40.44%) | 144(27.38%) | 370(49.66%) | <0.0001 |
| P_2_Y_12_ inhibitor, n (%) | 333(26.20%) | 74(14.07%) | 259(34.77%) | <0.0001 |
| Statin therapy, n (%) | 779(61.29%) | 230(43.73%) | 549(73.69%) | <0.0001 |
| PCSK9i therapy, n (%) | 6(0.47%) | 1(0.19%) | 5(0.67%) | 0.218 |
| LVEF, % | 61.90±12.57 | 62.62±14.60 | 61.39±10.90 | 0.0046 |
| FAI, HU | -79.10±7.79 | -80.23±7.97 | -78.30±7.57 | <0.0001 |
| IR indices |  |  |  |  |
| TyG index | 8.69±0.64 | 8.62±0.61 | 8.74±0.66 | 0.0006 |
| TyG-BMI | 224.13±146.05 | 217.05±114.07 | 229.12±164.83 | <0.0001 |
| AIP | 0.41±0.31 | 0.38±0.32 | 0.43±0.31 | 0.0005 |
| METS-IR | 39.34±25.51 | 38.13±18.65 | 40.20±29.39 | 0.0001 |

Data are presented as mean ± standard deviation (SD) or number (percentage), as appropriate. BMI, body mass index; PCSK9i, proprotein convertase subtilisin/kexin type 9 inhibitor; LVEF, left ventricular ejection fraction; FAI, fat attenuation index; HU, Hounsfield unit; IR, insulin resistance; TyG, triglyceride–glucose index; AIP, atherogenic index of plasma; METS-IR, metabolic score for insulin resistance.


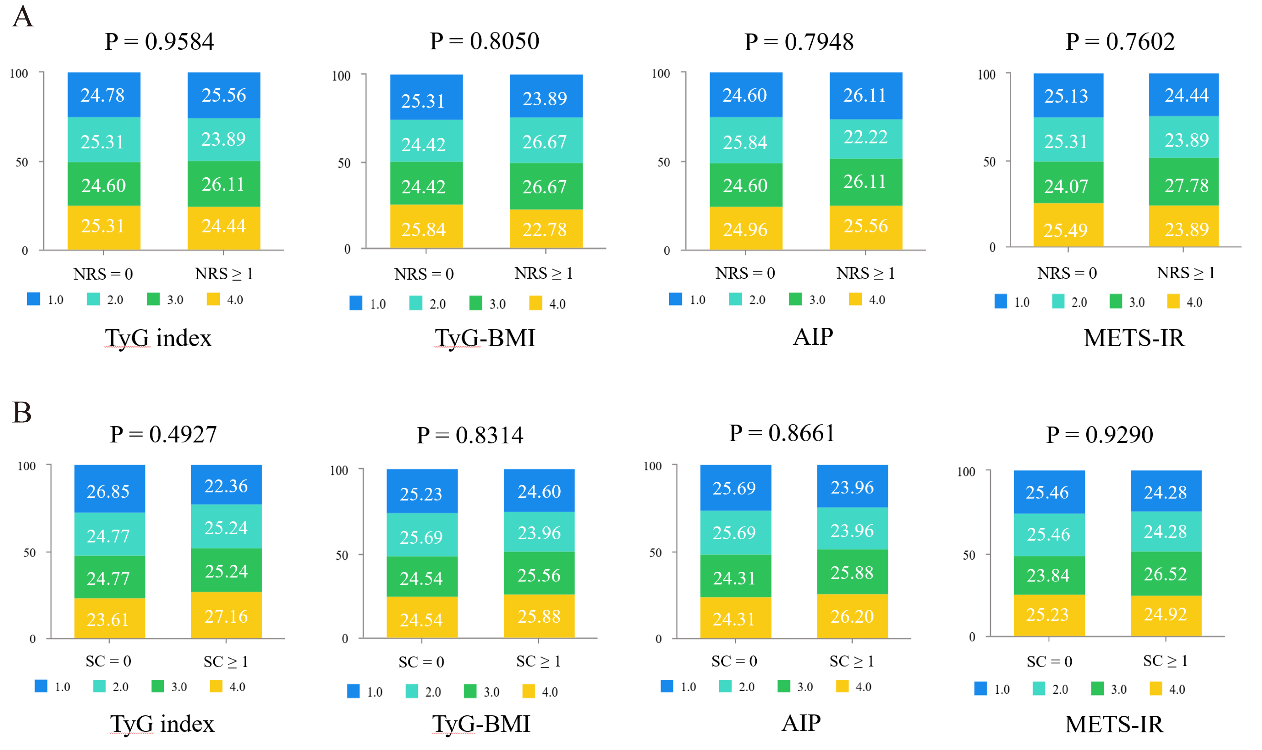


Supplemental Figure1 Distribution of NRS and SC according to IR indices

A. NRS features were not significantly different according to IR indices. B. SC features were not significantly different according to IR indices. SC, Spotty calcification; NRS, napkin-ring sign; TyG index, triglyceride-glucose index; TyG-BMI, TyG-body mass index; AIP, atherogenic index of plasma; METS-IR, metabolic score for insulin resistance.


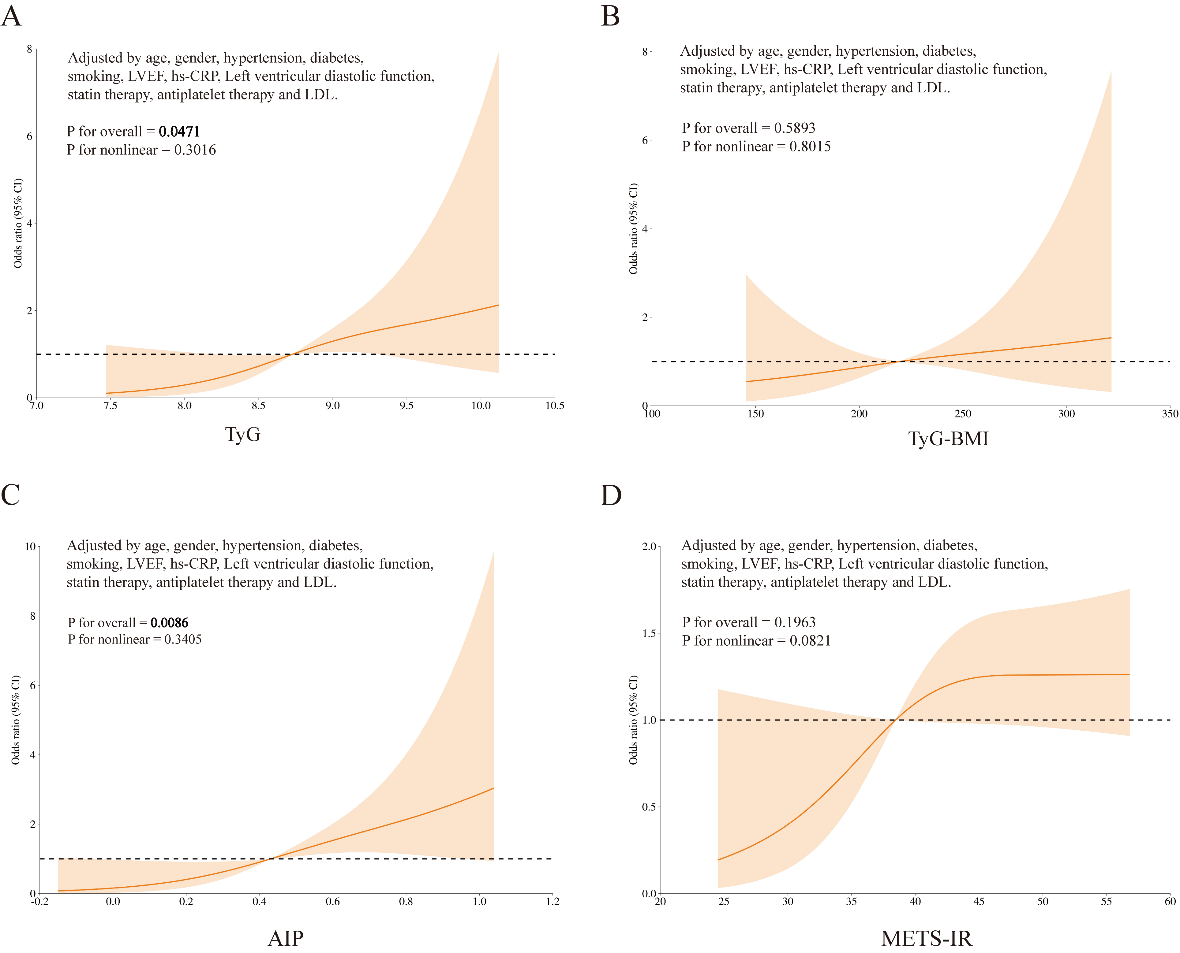


Supplemental Figure2 Restricted Cubic Spline Analyses of the Association Between IR Indices and MACE

A. RCS analysis of TyG and MACE; B. RCS analysis of TyG-BMI and MACE; C. RCS analysis of AIP and MACE; D. RCS analysis of METS-IR and MACE. TyG index, triglyceride-glucose index; TyG-BMI, TyG-body mass index; AIP, atherogenic index of plasma; METS-IR, metabolic score for insulin resistance; LVEF, left ventricular ejection fraction; hs-CRP, hypersensitive C reactive protein; LDL, low-density lipoprotein.
